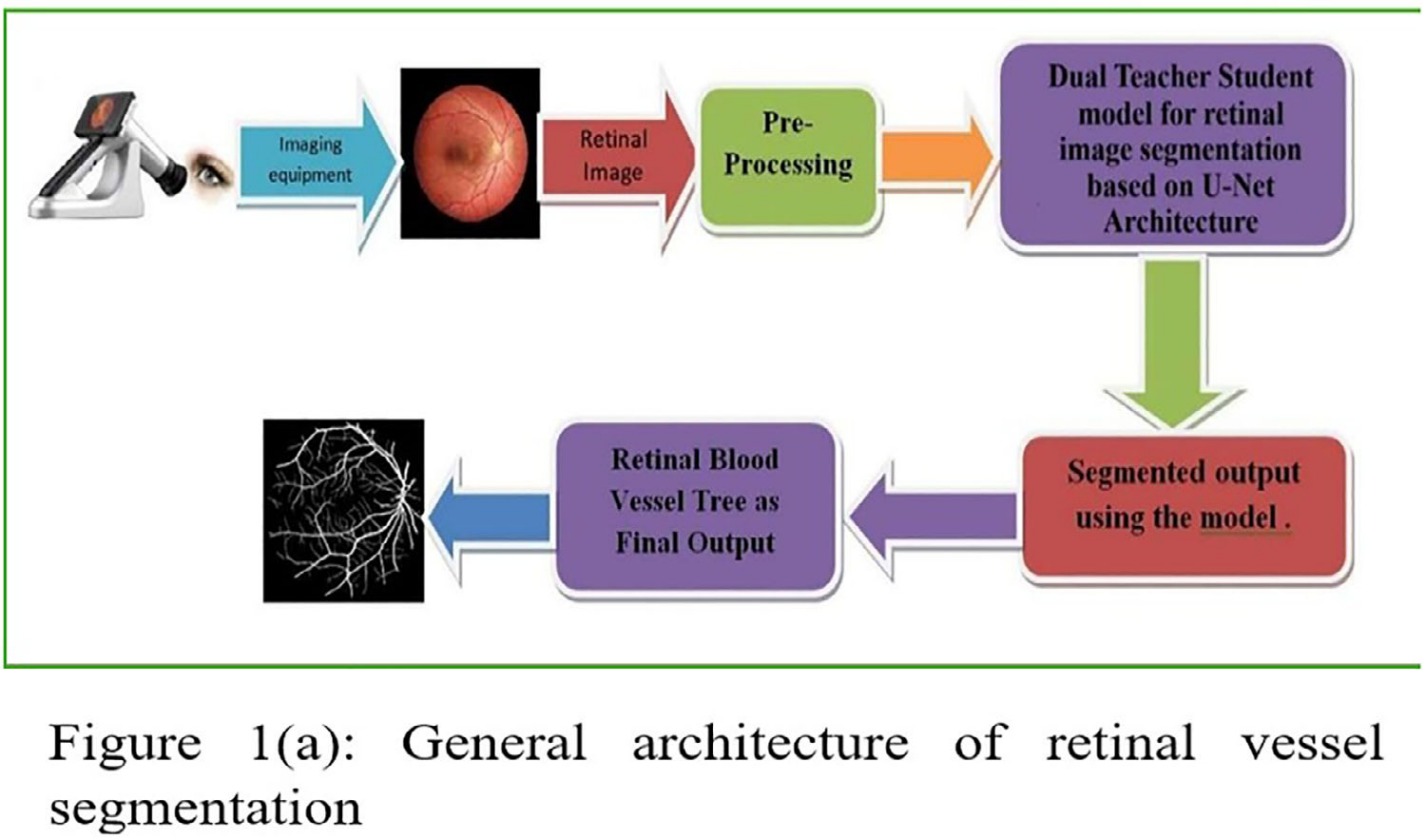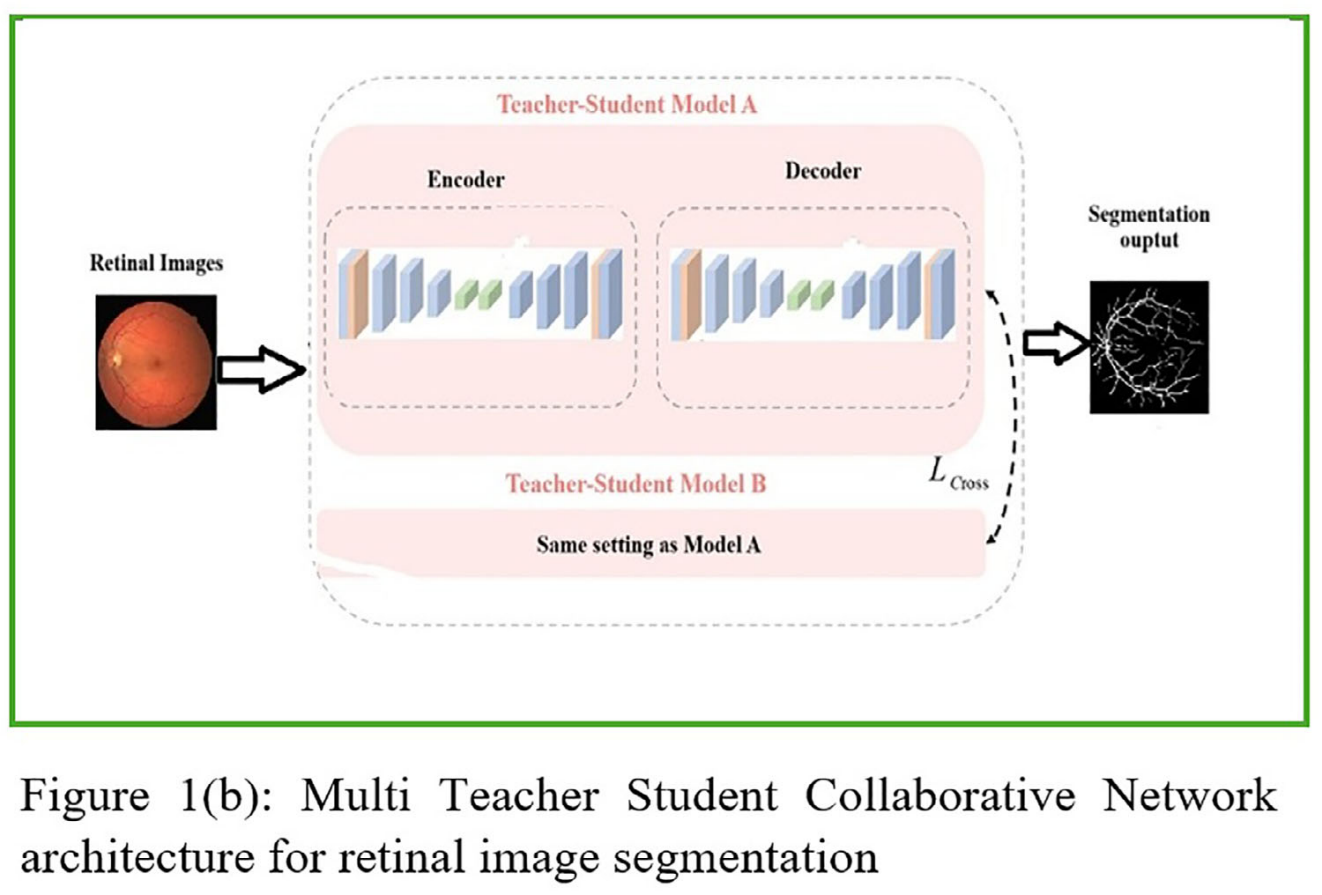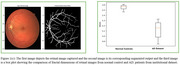# Analyzing the retinal vascular tree structure in Alzheimer's disease patients using fractal dimensions from retinal images

**DOI:** 10.1002/alz70856_096964

**Published:** 2025-12-24

**Authors:** Ansar A, Athira B, Rubell Marion Lincy G, Rakesh Shiradkar, Mangilal Agarwal, Sunu Mathew, Robert Mathew, Ancy R S

**Affiliations:** ^1^ Indian Institute of Information Technology, Kottayam, Kottayam, Kerala, India; ^2^ Indiana University, Indianapolis, IN, USA; ^3^ Indiana University School of Medicine, Indianapolis, IN, USA; ^4^ Sree Mookambika Institute of Medical Sciences, Kanyakumari, Tamil Nadu, India; ^5^ Ananthapuri Hospitals and Research Institute, Thiruvananthapuram, Kerala, India

## Abstract

**Background:**

Retinal vascular segmentation is an essential procedure in the automated analysis of fundus images for the screening and diagnosis of various diseases including Alzheimer's disease. This study presents a unique framework that employs semi‐supervised domain adaption and Multi Teacher‐Student (MTSC) Collaboration for Retinal Image Segmentation and calculation of Fractal Dimension (FD).

**Method:**

We have utilized a public dataset DRIVE for the development of our model. Also, we have collected the retinal images of 26 Alzheimer's Disease patients (48 images) and 28 normal controls (54 images) using a Non‐Mydriatic Retinal camera. The general architecture for segmentation is shown in Figure 1a. Since the retinal vasculature has a tree like structure which behaves like a fractal, it's essential to extract them from the retinal image, in order to perform fractal analysis. In this study, we present Multi Teacher Student (MTSC)˰architecture shown in Figure 1b which makes use of collaborative attention on several scales and dual supervision between teacher and students. After extracting the blood vessels, we have calculated the fractal dimensions of retinal images.

**Result:**

The proposed approach, which was assessed on the datasets comprising clinical images of the retina, demonstrates exceptional performance, achieving accuracy, IoU, AUC and F1‐Score values as 0.9786, 0.8406, 0.9206, and 0.8688 respectively. The output of segmentation is shown in Figure 1c. Also, we have calculated the fractal dimensions of both normal controls and AD patients. The Fractal dimensions (FD) of normal controls vary from 1.46 to 1.60 and FD of AD patients ranges from 0.84 to 1.32 shown in Figure 1d. Table 1e shows the vessel extraction method and the ranges of FD of retinal images from various dataset. The FD showed statistically significant difference between normal controls and Alzheimer's disease patients (*p* = 0.000048). The fractal dimension of AD patients are significantly lower than the FD of normal controls (*p* <0.05).

**Conclusion:**

In this study we can conclude that the FD from the retinal vasculature can be used as one of the bio markers for predicting Alzheimer's disease. The study reveals that the FD of the AD patients are comparatively lesser than that of the normal control.